# Proteomic Profiling of SGLT-2 Inhibitor Canagliflozin in a Swine Model of Chronic Myocardial Ischemia

**DOI:** 10.3390/biomedicines12030588

**Published:** 2024-03-06

**Authors:** Dwight D. Harris, Sharif A. Sabe, Mark Broadwin, Christopher Stone, Cynthia Xu, Jiayu Hu, Meghamsh Kanuparthy, M. Ruhul Abid, Frank W. Sellke

**Affiliations:** Division of Cardiothoracic Surgery, Department of Surgery, Cardiovascular Research Center, Rhode Island Hospital, Alpert Medical School of Brown University, Providence, RI 02903, USA; ddharris@bidmc.harvard.edu (D.D.H.); ssabe@bidmc.harvard.edu (S.A.S.); mbroadwin@lifespan.org (M.B.); christopher_stone@brown.edu (C.S.); cxu2@lifespan.org (C.X.); jiayu_hu1@brown.edu (J.H.); meghamsh_kanuparthy@brown.edu (M.K.); ruhul_abid@brown.edu (M.R.A.)

**Keywords:** sodium–glucose cotransporter-2 inhibitors, proteomic, swine, chronic myocardial ischemia, canagliflozin, cardiac index, metabolism, redox

## Abstract

Background: Sodium–glucose cotransporter-2 (SGLT2) inhibitors are known to be cardioprotective independent of glucose control, but the mechanisms of these benefits are unclear. We previously demonstrated improved cardiac function and decreased fibrosis in a swine model of chronic myocardial ischemia. The goal of this study is to use high-sensitivity proteomic analyses to characterize specific molecular pathways affected by SGLT-2 inhibitor canagliflozin (CAN) therapy in a swine model of chronic myocardial ischemia. Methods: Chronic myocardial ischemia was induced in sixteen Yorkshire swine via the placement of an ameroid constrictor to the left circumflex coronary artery. After two weeks of recovery, swine received either 300 mg of CAN daily (*n* = 8) or a control (*n* = 8). After five weeks of therapy, the group of swine were euthanized, and left ventricular tissue was harvested and sent for proteomic analysis. Results: Total proteomic analysis identified a total of 3256 proteins between the CAN and control groups. Three hundred and five proteins were statistically different. This included 55 proteins that were downregulated (*p* < 0.05, fold change <0.5) and 250 that were upregulated (*p* < 0.05, fold change >2) with CAN treatment. Pathway analysis demonstrated the upregulation of several proteins involved in metabolism and redox activity in the CAN-treated group. The CAN group also exhibited a downregulation of proteins involved in motor activity and cytoskeletal structure. Conclusions: In our swine model of chronic myocardial ischemia, CAN therapy alters several proteins involved in critical molecular pathways, including redox regulation and metabolism. These findings provide additional mechanistic insights into the cardioprotective effects of canagliflozin.

## 1. Introduction

Ischemic cardiovascular disease (CVD) remains a leading cause of morbidity and mortality both in the United States and worldwide. Beyond its effects on individual patients, this disease entity also imposes a substantial burden and cost on the healthcare system at large [1,2,3]. While medical therapy, including the use of antiplatelet agents, beta-blockers, and statins, has made significant strides in managing ischemic CVD, there are limitations to the efficacy of these agents, and a considerable number of patients are refractory to maximal medical therapy [1,2]. Furthermore, a significant number of patients are not candidates for standard revascularization via coronary artery bypass grafting or percutaneous coronary intervention. Research and development efforts are, thus, pivotal in uncovering innovative approaches to tackle ischemic CVD with a focus on interventions that can mitigate the underlying causes and improve overall cardiovascular function for patients with limited or no procedural options.

Given this, interest has been growing in recent years in the adjunctive use of novel antidiabetic medications in the treatment of coronary artery disease. Sodium–glucose cotransporter-2 (SGLT-2) inhibitors represent a relatively recently approved class of medications that have significantly impacted the management of type 2 diabetes mellitus as well as coronary artery disease [4]. SGLT-2 inhibitors function in their capacity as antidiabetic agents to decrease glucose reabsorption in the proximal convoluted renal tubules but have also been shown to improve cardiovascular outcomes in human and animal models. Although this was originally discovered through the performance of cardiovascular safety trials enrolling diabetic patients, it was subsequently demonstrated to apply to all patients regardless of their diabetic status [5].

Accordingly, data from both prospective and retrospective clinical studies showed a decrease in cardiac events and cardiovascular mortality in diabetic patients treated with SGLT2 inhibitors [6,7,8,9,10,11]. SGLT-2 inhibitors also decrease cardiovascular mortality and hospital readmission in patients with congestive heart failure (CHF) independently of diabetic status. Given this growing and substantial body of evidence, including several meta-analyses supporting the use of SGLT-2 inhibitors in cardiovascular disease, the United States Food and Drug Administration has expanded indications for the use of SGLT2 inhibitors as a first-line therapy and a component of the goal-directed medical treatment of CHF; specifically, SGLT-2 inhibitors have gained a 1A recommendation for use in the treatment of heart failure with reduced ejection fraction [12,13].

Even though the clinical literature to support the use of SGLT-2 inhibitors is increasing, the mechanism through which these agents act on the myocardium to produce improved outcomes over the long term remains poorly understood [14,15,16]. Small animal models using rodents have demonstrated increased cardiac output, decreased myocardial remodeling, reduced oxidative stress, and improved vascular reactivity in both acute ischemia and reperfusion injury and in healthy myocardium [9,17,18]. Owing to the heterogeneity between small animal models and the patients they are designed to represent, however, these models are unable to fully recapitulate the features of adult CVD and, thus, fall short of the explanatory power needed to fully understand the effects of SGLT-2 inhibitors in clinical practice.

To increase our knowledge of how SGLT-2 inhibition improves cardiac function, our lab chose to extensively study SGLT2 inhibitors in chronic myocardial ischemia using a swine model of chronic myocardial ischemia [19]. We previously demonstrated several remarkable changes with this model, including increased myocardial function, improved coronary perfusion, and decreased myocardial fibrosis [19]. We also began to elucidate the molecular underpinnings of these functional benefits, having already demonstrated modulations in angiogenic signaling, insulin signaling, and myocardial inflammation following treatment with the SGLT-2 inhibitor canagliflozin (CAN) [20,21]. The objective of this study is to expand our understanding of SGLT-2 inhibition in the myocardium using CAN treatment in the context of our swine model of chronic myocardial ischemia and draw on total proteomic analysis as the most sensitive method for this purpose.

## 2. Methods

### 2.1. Animal Model

This study is a continuation of our previously published swine cohort that received a regular diet [19]. Sixteen 11-week-old *Yorkshire swine* (Cummings School of Veterinary Medicine of Tufts University Farm, North Grafton, MA, USA) arrived at our facility at 9 weeks of age. After a brief acclimation period, all animals underwent ameroid constrictor (Research Instruments SW, Escondido, CA, USA) placement on the left coronary circumflex artery (LCx) through a left-sided mini-thoracotomy. After ameroid placement, the swine underwent a two-week recovery to allow for the closure of the ameroid device, as this allows our experimental model to faithfully represent the initiation of therapy in the setting of established disease. After two weeks elapsed, all animals were assigned to either a vehicle (control, *n* = 8, female = 3, male = 5) or 300 mg of CAN daily (*n* = 8, female = 4, male = 4). Five weeks of therapy were administered in accordance with the temporality of effect emergence in humans. Finally, swine underwent a terminal harvest procedure, entailing functional studies, the collection of the ischemic myocardium, and perfusion analysis.

### 2.2. Humane Animal Care

This study was approved by The Rhode Island Hospital Institutional Animal Care and Use Committee (Protocol #505821, 23 November 2021). Animals were cared for in compliance with the Principles of Laboratory Animal Care and the Guide for the Care, Declaration of Helsinki, and Use of Laboratory Animals [19].

### 2.3. Ameroid Constrictor

Anesthesia and preoperative care were administered as previously reported [19]. The swine was placed on the operating room table in a modified right lateral decubitus position and prepped in a sterile fashion with betadine. Preoperative antibiotics and aspirin were administered in accordance with our previously published protocol to minimize the risk of infection and coronary thrombosis, respectively [19]. A left mini-thoracotomy was performed in the second intercostal space. The pericardium was opened and secured to the chest wall with a 2-0 silk suture. The left atrium was visible directly below the pericardiotomy and was retracted with a silk suture to expose the underlying LCx. The LCx was dissected free from the surrounding epicardial fat and other connective tissue using a combination of blunt and sharp dissection and was isolated at its takeoff from the left main coronary artery proximal to the first obtuse marginal vessel. The swine was heparinized (80 IU/kg), and the LCx was secured with a vessel loop. The area at risk for ischemia was then mapped by injecting 5 mL of gold microspheres (BioPal, Worcester, MA, USA) into the left atrial appendage while occluding the LCx with the vessel loop, as this resulted in the selective distribution of the microsphere to the coronary circulation apart from the LCx territory. Using the vessel loop for leverage on the artery as needed, the ameroid constrictor was then placed on the LCx. Nitroglycerin was given topically to offset coronary vasospasm secondary to arterial manipulation. The chest was closed in a layered fashion with an absorbable suture, as previously reported [19]. Postoperative pain control was administered and sustained analogously, as previously reported [19].

### 2.4. Tissue Harvest

Anesthesia and preoperative care were administered, as previously reported [19]. The swine was placed in a supine position and prepped in a sterile fashion with betadine. A median sternotomy was performed to adequately expose the left ventricle, left atrium, and right atrium. The right femoral artery was exposed with a groin cutdown [19]. The swine was heparinized (80 IU/kg) prior to arterial instrumentation. The artery was then canulated with a 7 French vascular sheath using the Seldinger technique [19]. One pressure catheter (Transonic, Ithaca, NY, USA) was advanced through the sheath into the aorta from the right femoral artery, after which a second pressure–volume catheter (Transonic, Ithaca, NY, USA) was inserted into the apex of the left ventricle, also using the Seldinger technique following ventriculotomy under direct visualization [19]. Hemodynamic data were recorded using these catheters and analyzed with LabChart software 8.1.28 (ADInstruments, Sydney, NSW, Australia) [19]. Upon the completion of physiologic measurement acquisition, anesthesia was deepened, and the heart was excised. Following excision, the heart was cut into 16 sections based on the position relative to the LCx, and the area of ischemia was confirmed by measuring the gold microsphere count as described above. The remaining tissue was flash-frozen in liquid nitrogen for molecular studies, including immunoblotting and proteomic analysis.

### 2.5. Proteomic Analysis

The proteomic analysis was performed, as previously reported by the proteomics core facility at the University of Massachusetts-Boston [22]. Ischemic myocardial tissue was processed, and the protein concentration of the homogenates was determined by the BCA assay, as previously reported [22]. Lysates were TMTpro-labeled and multiplexed, as previously reported. The multiplexed samples were then fractionated into 10 fractions via reversed-phase high-performance liquid chromatography (Thermo UltiMate 3000, Waltham, MA, USA) as previously reported [22]. The fractions were then analyzed via nano-liquid chromatography-mass spectrometry on an EASY-1200 nano-liquid chromatography apparatus coupled online to an orbitrap Fusion Lumos, where quantification was performed [22]. Thermo Fisher’s Proteome Discoverer (Waltham, MA, USA) was used for processing with a false discovery rate of 0.1%. Data were normalized to the total peptide amount. Please see our prior manuscript for supplementary material, including detailed proteomic methods [22].

### 2.6. Immunoblotting

Ischemic myocardial tissue was aliquoted and then lysed using an RIPA Lysis and Extraction Buffer and the Halt Protease Inhibitor Cocktail (Thermo Fisher Scientific, Waltham, MA, USA), as previously reported [20]. The lysate (40 μg) was run on a 4% to 12% Bis-Tris gel (Thermo Fisher Scientific, Waltham, MA, USA) and transferred to a nitrocellulose membrane (Thermo Fisher Scientific, Waltham, MA, USA). Membranes were incubated for 24 h at 4 degrees Celsius with primary antibodies in 3% bovine serum albumin in tris-buffered saline with tween (Boston Bioproducts, Milford, MA, USA), as previously described (see Table 1 for a list of antibodies utilized in this experiment) [20]. Horseradish peroxidase-linked secondary antibodies of the mouse or rabbit (Cell Signaling, Danvers, MA, USA), as appropriate, were incubated for 1 h at room temperature. Imaging was performed using a ChemiDoc Imaging System (Bio-Rad, Hercules, CA, USA) and using the enhanced chemiluminescence Western Blotting Substrate (Thermo Fisher Scientific, Waltham, MA, USA) as a developing agent. Repeated probing was performed by stripping membranes with a Restore PLUS Western Blot Stripping Buffer (Thermo Fisher Scientific, Waltham, MA, USA). Finally, data were analyzed using Image J software 1.54i (National Institutes of Health, Bethesda, MD, USA).

### 2.7. Statistics

Data from immunoblot analysis are presented as the mean fold change compared to the average control with standard deviations. Immunoblot data were analyzed with the two-tailed Student’s *t* test. All Immunoblot analyses were conducted with Prism 10 (GraphPad Software, San Diego, CA, USA). Probability values less than 0.05 were predefined as significant.

For proteomic analysis, significant changes in protein expression were defined as fold changes greater than 2 or less than 0.5 with an unadjusted *p*-value less than or equal to 0.05. Pathway analysis was performed using ShinyGo 0.76 (South Dakota State University, Brookings, SD, USA) software [23].

## 3. Results

### 3.1. Cardiac Index

**Figure 1:**
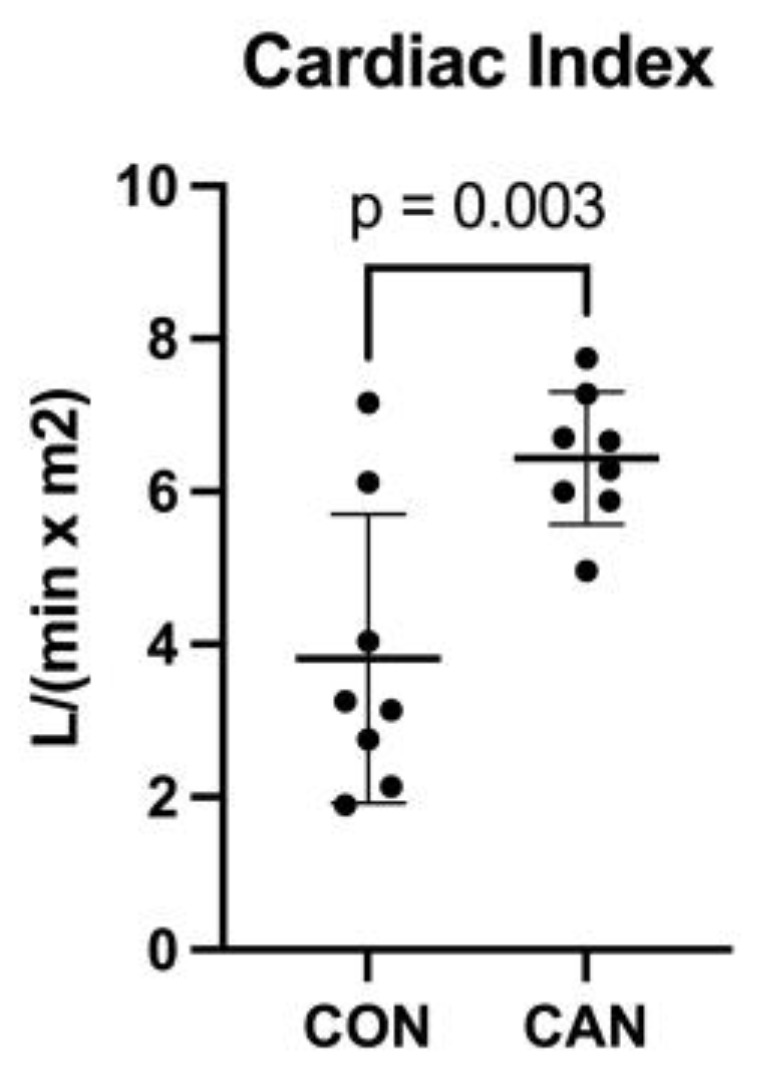
Cardiac index. The cardiac index was significantly increased in the canagliflozin (CAN) group compared to the control (CON).

The functional data showed a significant increase in cardiac index in the CAN group compared to the CON group (*p* = 0.003, Figure 1).

### 3.2. Total Proteomics and Pathway Analysis

**Figure 2:**
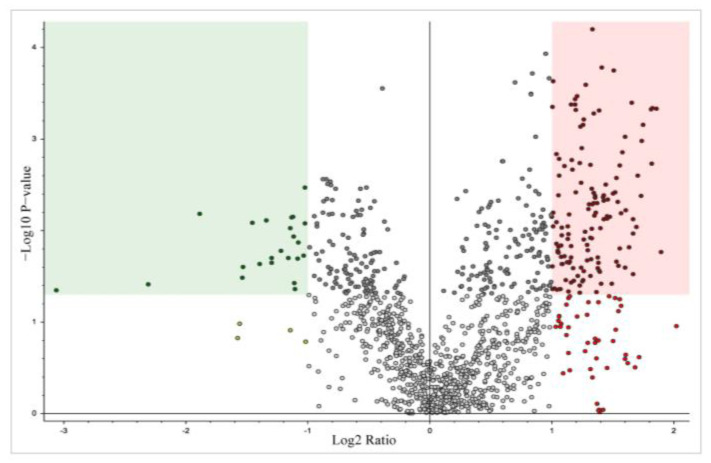
Volcano plot. Total proteomic analysis revealed 3256 common proteins between the control and canagliflozin groups. Of these proteins, 305 were statistically different, of which 250 were upregulated (*p* < 0.05, fold change >2), and 55 were downregulated (*p* < 0.05, fold change <0.5) with the administration of CAN. The fold change was calculated as the ratio of protein expression in the canagliflozin group compared to that of the control group. Data points in the green box on the upper left corner of the diagram are significantly downregulated; data points in the red box on the upper right corner of the diagram are significantly upregulated.

**Figure 3:**
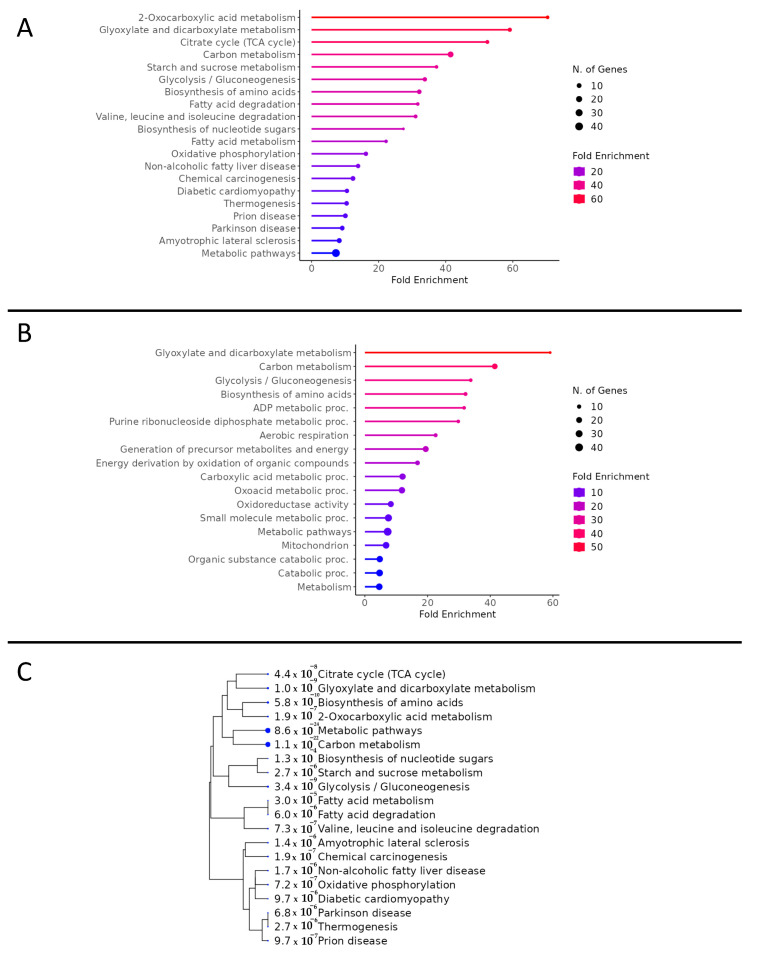
Pathway analysis of increased proteins. (**A**) KEGG pathway analysis of the upregulated proteins demonstrated an increase in pathways related to glucose metabolism, fatty acid metabolism, and oxidative phosphorylation in the canagliflozin group. (**B**) Universal pathway analysis also identified changes in glucose metabolism and mitochondrial components in the canagliflozin group. (**C**) A tree diagram was generated to display the clustering of upregulated proteins using KEGG analysis. The size of the blue dot to the left of the pathway designation indicates the relative quantity of upregulated genes in the pathway.

**Figure 4:**
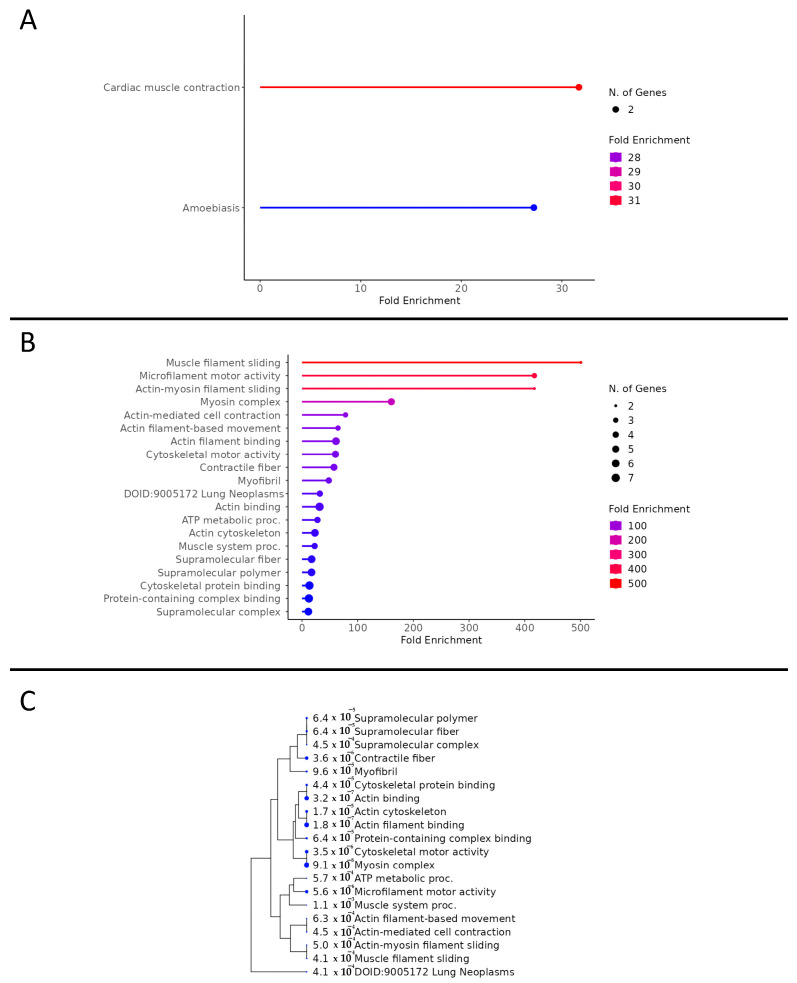
Decreased pathway analysis. (**A**) This figure displays a KEGG analysis of decreased proteins in cardiac muscle contraction in the canagliflozin group compared to the control. (**B**) Universal pathway analysis showed decreases in muscle filaments, myosin complexes, and other cytoskeletal proteins in the canagliflozin group compared to the control. (**C**) A tree diagram was generated to display the clustering of downregulated proteins using universal pathway analysis. The size of the blue dot to the left of the pathway designation indicates the relative quantity of upregulated genes in the pathway.

Total proteomic analysis revealed 3256 proteins in common between the CON and CAN groups. Of these proteins, 305 were statistically different, of which 250 were upregulated (*p* < 0.05, fold change >2), and 55 were downregulated (*p* < 0.05, fold change <0.5) with the CAN group (Figure 2). The KEGG pathway analysis of the upregulated proteins demonstrated an increase in pathways related to glucose metabolism, fatty acid metabolism, and oxidative phosphorylation in the CAN group (Figure 3A). Universal pathway analysis also identified changes in glucose metabolism and mitochondrial components (Figure 3B). The KEGG analysis of the decreased proteins demonstrated changes in proteins related to cardiac muscle contraction in the CAN group compared to the controls (Figure 4A). The universal analysis showed decreases in muscle filaments, myosin complexes, and other cytoskeletal proteins in the CAN group compared to the controls (Figure 4B).

### 3.3. Metabolism

**Figure 5:**
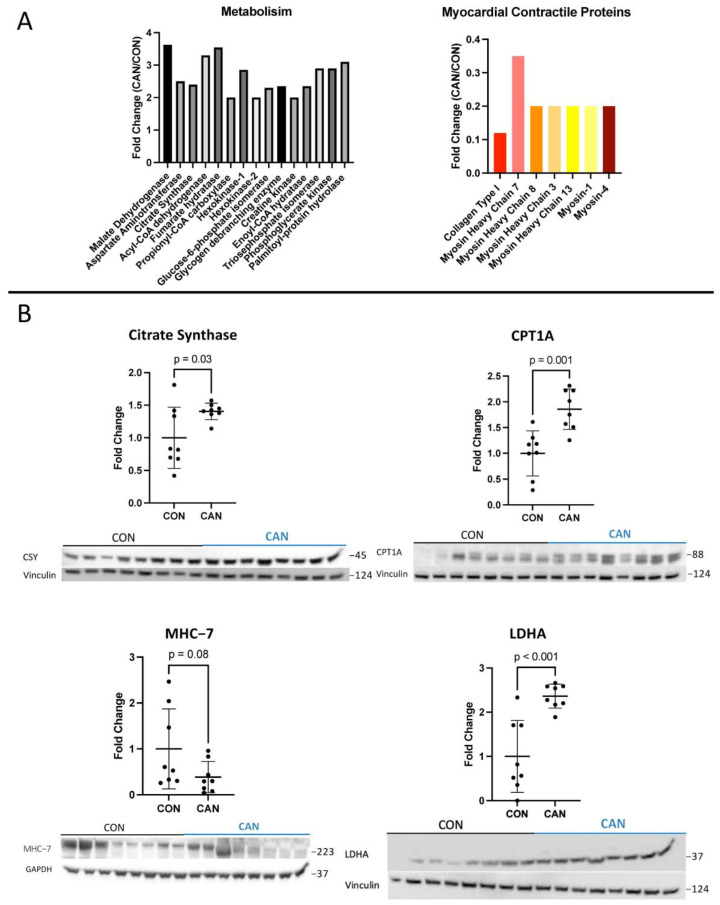
Metabolism and myocardial contractility: (**A**) Proteomic analysis showed a significant increase in metabolic proteins and proteins involved in myocardial contractility in the canagliflozin (CAN) group compared to the control. Affected proteins included malate dehydrogenase, aspartate aminotransferase, citrate synthase, acyl-CoA dehydrogenase, fumarate hydratase, propionyl-CoA carboxylase, hexokinase-1, hexokinase-2, glucose-6-phosphate isomerase, glycogen debranching enzyme, creatine kinase, enoyl-CoA hydratase, triosephosphate isomerase, phosphoglycerate kinase, palmitoyl-protein hydrolase, collagen type I, myosin heavy chain 7, myosin heavy chain 8, myosin heavy chain 3, myosin heavy chain 13, myosin 1, and myosin 4. (**B**) Immunoblotting validation showed a significant increase in CPT1A, lactate dehydrogenase A (LDHA), and citrate synthase (CSY), as well as a trend towards increased myosin heavy chain 7 (MHC-7).

Proteomic analysis showed a significant increase in metabolic proteins in the CAN group, including malate dehydrogenase, aspartate aminotransferase, citrate synthase, acyl-CoA dehydrogenase, fumarate hydratase, propionyl-CoA carboxylase, hexokinase-1, hexokinase-2, glucose-6-phosphate isomerase, glycogen debranching enzyme, creatine kinase, enoyl-CoA hydratase, triosephosphate isomerase, phosphoglycerate kinase, and palmitoyl-protein hydrolase (Figure 5A). This was validated by immunoblotting, showing a significant increase in CPT1a, lactate dehydrogenase A, and citrate synthase (all *p* < 0.05, Figure 5B).

### 3.4. Myocardial Contractility

Proteomic analysis showed a significant decrease in several myocardial contractile proteins in the CAN group, including collagen type I, myosin heavy chain 7, myosin heavy chain 8, myosin heavy chain 3, myosin heavy chain 13, myosin 1, and myosin 4 (Figure 5A). This was supported by a trend toward decreased myosin heavy chain 7 on immunoblotting (*p* = 0.08, Figure 5B).

### 3.5. Oxidative Phosphorylation

**Figure 6:**
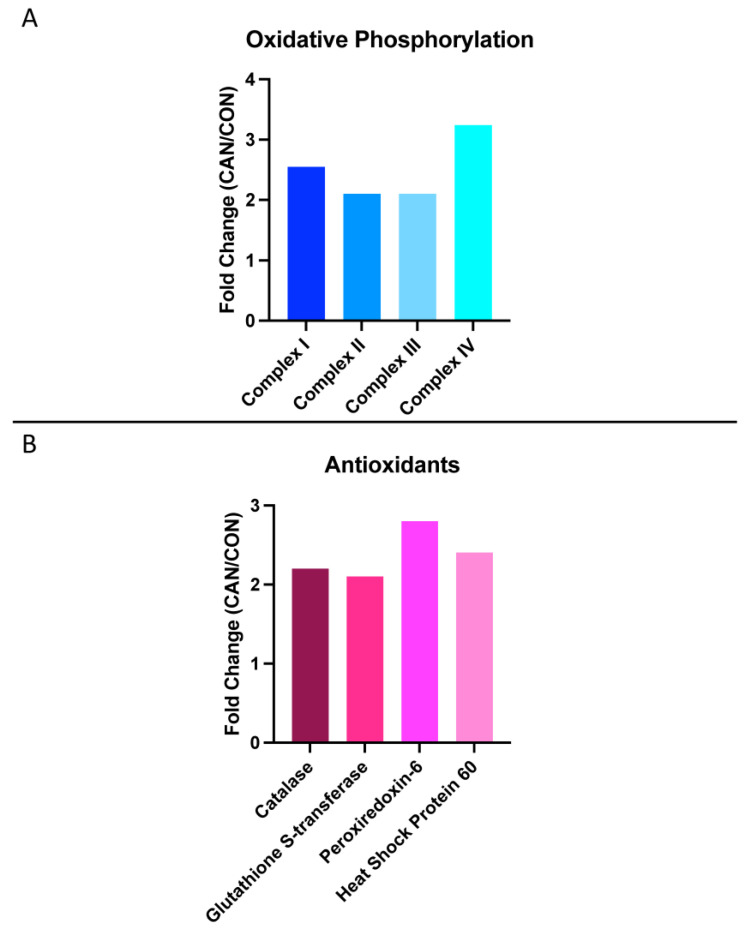
Oxidative phosphorylation and antioxidants. (**A**) Proteomic analysis showed a significant increase in mitochondrial complexes in the canagliflozin group, including Complex I, Complex II, Complex III, and Complex IV. (**B**) The canagliflozin group exhibited an increase in several antioxidants on proteomic analysis as well, including catalase, glutathione-s-transferase, peroxiredoxin-6, and heat shock protein 60.

Proteomic analysis revealed a significant increase in mitochondrial complexes in the CAN group, including Complex I, Complex II, Complex III, and Complex IV (Figure 6A).

### 3.6. Antioxidants

The CAN group exhibited an increase in several antioxidants on proteomic analysis, including catalase, glutathione-s-transferase, peroxiredoxin-6, and heat shock protein 60 (Figure 6B).

## 4. Discussion

The treatment of advanced heart disease is evolving to include novel medical therapies, such as SGLT-2 inhibitors and glucagon-like peptide-1 receptor agonists [13,24]. SGLT-2 inhibitors are of particular interest, as they have been shown to decrease heart disease mortality and symptom burden in numerous large randomized controlled trials, leading to the incorporation of these agents into routine clinical practice for patients with CHF. The pathways affected by these drugs and responsible for their efficacy in the context of the ailing heart, however, remain largely unknown. We previously demonstrated increased myocardial perfusion, enhanced ventricular relaxation, and a strong trend towards increased cardiac output using our validated large animal mode of chronic myocardial ischemia [19]. We also identified changes in myocardial fibrosis, inflammation, and oxidative stress in our prior work, which was limited to the use of immunoblotting and constrained by the low-throughput nature of this technique [19,20,21]. This study represents a significantly more sophisticated follow-up investigation into the molecular changes produced by CAN and, therefore, provides a far greater resolution in our capacity to characterize these changes and associate them with the clinical efficacy of SGLT-2 inhibition.

In this study, we again demonstrated increased cardiac function with CAN by showing an increase in the cardiac index, which further validates our previous findings, suggesting increased left ventricular function and cardiac output. This increase in the cardiac index is an important benefit of CAN treatment and may play a role in the decrease in heart failure mortality and hospitalizations previously reported with SGLT-2 inhibitors. The proteomics analysis from our study revealed changes in several pathways that likely play a role in this increase in the cardiac index as well as in our other prior findings.

The results of our study demonstrate a significant increase in markers of metabolism in glycolysis, the citric acid cycle, and fatty acid oxidation in CAN-treated animals compared to their control counterparts. In particular, we saw significant increases in glycolysis-related proteins, including hexokinase 1, hexokinase 2, and glucose-6-phosphate isomerase. This was coupled with an increase in citric acid cycle enzymes, including an increase in malate dehydrogenase, aspartate aminotransferase, citrate synthase, and acyl-CoA dehydrogenase. These results suggest that CAN treatment produces an increase in glycolysis that likely culminates in downstream increases in both the citric acid cycle and oxidative phosphorylation. These changes are additionally accompanied by an increase in fatty acid metabolism, as seen through changes in palmitoyl-protein hydrolase, enoyl-CoA hydratase, and CPT1A. Myocardial ischemia is known to lead to the significant dysregulation of myocardial metabolism, typically manifested as a shift away from aerobic adenosine triphosphate formation in favor of glycolysis and a disruption in fatty acid metabolism [25,26,27,28]. This suggests that CAN induces a shift back to aerobic metabolism and promotes fatty acid utilization. This shift in metabolism was validated by an increase in oxidative phosphorylation complexes in the CAN group compared to the control. It is established that myocardial ischemia results in decreased oxidative phosphorylation; taken in conjunction with our results, this implies that CAN may exert its favorable effects through the normalization of myocardial metabolism [29]. This improvement in myocardial energetics may, in turn, account for the hemodynamic augmentation observed through the improved cardiac index found in treated animals.

Oxidative stress plays an important role in the maladaptive remodeling of myocardial ischemia [30]. SGLT2 inhibitors have been shown to ameliorate inflammation and oxidative stress in multiple organ systems [31,32,33]. The results of our study demonstrate a significant increase in myocardial antioxidants, including catalase, peroxiredoxin-6, glutathione S-transferase, and heat shock protein 60. We previously showed that CAN reduces oxidative stress, but this study provides insight into the upregulation of specific myocardial antioxidants by CAN [19]. This increase in antioxidants likely contributes to the known reduction in fibrosis seen with CAN [19].

As described above, however, CAN additionally appears to produce an ostensibly counterintuitive decrease in several myocardial contractile proteins. In particular, we noted decreases in collagen type I and myosin heavy chain. This was likely due to the nature of the proteins considered contractile in the analysis, as many of these play multiple roles in vivo. For example, the decrease in collagen type I could be related to our previously published findings on decreased fibrosis [34,35]. Similarly, the decrease in myosin heavy chain could signify a reduction in heart hypertrophy in the CAN group compared to the control [36,37]. These findings may provide some mechanistic explanation of the improved diastolic function seen with canagliflozin treatment in the setting of chronic myocardial ischemia [19].

In summary, proteomic analysis showed that CAN results in changes in several molecular pathways in the myocardium, including increased myocardial metabolism, augmented oxidative phosphorylation, and decreased oxidative stress. These results help characterize the mechanism of CAN in chronic ischemic heart disease and, thus, identify directions for further research in patients treated with SGLT-2 inhibitors.

Its robust methodology notwithstanding, this study possesses some limitations meriting mention. First, given the nature of large animal work and the cost of high-sensitivity proteomics, the sample size is relatively small, comprising a total of 16 subjects. Additionally, this study utilizes mixed-sex animals, but given that it contains only three females, it is not designed for sex-specific comparisons. Additionally, this study only uses one SGLT-2 inhibitor, CAN, and the findings might vary with the use of other SGLT-2 inhibitors. Finally, and perhaps most importantly, this study is limited by the proteomic methods employed, as different extraction and running methods can generate different results. For instance, the proteomics methods used in this paper do not identify phosphorylated proteins, and phosphorylation plays an important role in the regulation of cellular function. In future work, we hope to employ larger sample sizes and expand the scope of the proteomic methods employed in this experiment to characterize the effects of CAN with even greater granularity.

## 5. Conclusions

CAN results in an increase in cardiac function in the setting of chronic myocardial ischemia. This increase in cardiac function is accompanied by increases in the proteins involved in myocardial metabolism, oxidative phosphorylation, and antioxidants within the ischemic myocardium. These findings provide additional mechanistic insights into the cardioprotective effects of the SGLT-2 inhibitor canagliflozin and provide targets for further clinical investigation.

## Figures and Tables

**Table 1:** Antibodies. Table 1 displays a list of all of the antibodies and their manufacturers used in this experiment.

Antibody Name	Company	Catalog Number
Citrate synthase	Cell Signaling	14309
CPT1a	Abcam	ab128568
Lactate dehydrogenase A	Cell Signaling	2012
GAPDH	Cell Signaling	5174
Myosin heavy chain 7	Proteintech	22280-1-AP
Vinculin	Cell Signaling	13901
Mouse secondary antibody	Cell Signaling	7076
Rabbit secondary antibody	Cell Signaling	7074

## Data Availability

All data are available on request to the corresponding author.
